# The Impact of Family Relationship on Serum 25-Hydroxyvitamin D (25OHD) Concentrations in Individuals Residing in Pune, India

**DOI:** 10.7759/cureus.71513

**Published:** 2024-10-15

**Authors:** Rubina Mandlik, Swapna Deshpande, Anuradha Khadilkar

**Affiliations:** 1 Growth and Pediatric Endocrinology, Hirabai Cowasji Jehangir Medical Research Institute, Pune, IND; 2 Health Sciences, Tampere University, Tampere, FIN; 3 Health Sciences, Savitribai Phule Pune University, Pune, IND

**Keywords:** 25ohd, 25ohd2, adults, children, family, intra-family correlation, siblings, vitamin d

## Abstract

Introduction: An understanding of the influence of family relationship on vitamin D concentrations could be useful in designing community-based strategies to improve vitamin D status. Hence, the aim of this study was to explore if family relationship had an impact on total serum 25-hydroxyvitamin D (25OHD) concentrations of individuals living in Pune, India.

Methods: This cross-sectional study included 104 families. Data collected included anthropometry (height and weight), body composition (bioelectric impedance), sunlight exposure (validated questionnaire), and blood samples for estimation of serum 25OHD_2_ and 25OHD_3_ by liquid chromatography-tandem mass spectrometry (LC-MS/MS) method. Total serum 25OHD was computed as the sum of the serum 25OHD_2_ and 25OHD_3_ concentrations. Linear mixed model analysis was employed to assess the impact of family on serum 25OHD_2_ and total 25OHD concentrations. The strength of the family-level component was assessed by calculating the intra-family correlation for each model separately for children and adults.

Results: More than half (63%) of the participants of the participants (235 adults, 118 children) had poor daily sunlight exposure (< 30 minutes). Around one-third, both adults and children, were found to be vitamin D deficient. Intra-family correlation was found to be higher in children than in adults for serum 25OHD_2_ (0.6 vs 0.4) and total serum 25OHD (0.5 vs 0.1) concentrations.

Conclusions: Our findings indicate that the likelihood of having similar vitamin D concentrations is higher among children as compared to adults in a family. This suggests a role played by genetic factors or possibly shared habits among children in a family, thus resulting in stronger correlations. This can facilitate the adoption of early preventive measures for treatment or prevention of vitamin D deficiency, even if one child among siblings is vitamin D insufficient.

## Introduction

Vitamin D is an essential fat-soluble vitamin with a primary role in musculoskeletal health. The major source of vitamin D is cutaneous photoproduction. India experiences an abundance of sunlight throughout the year. Still, around 75% of Indians are found to have insufficient vitamin D concentrations. Traditional clothing practices, increased urbanization, and decreased daily sun exposure due to increased time spent indoors could be some of the reasons for the high prevalence of vitamin D insufficiency in India [1-3]. The development of low bone mass in adulthood and the greater risk of osteomalacia and osteoporosis with advancing age (50 years and above) can largely be attributed to vitamin D deficiency (VDD) during bone development in the early years of life [4].

Family relationship may have an influence on vitamin D concentrations due to shared genetics and lifestyle habits [5]. However, this has not been estimated among Indians. The knowledge of the influence of family relationship on vitamin D concentrations could be useful in designing community-based strategies to improve vitamin D status [6]. Hence, we planned this study with the aim of exploring if family relationship had an impact on total serum 25-hydroxyvitamin D (25OHD) concentrations of individuals living in Pune, India. This article was previously presented as a poster at the 11^th^ International Conference on Children's Bone Health on June 24, 2024 [7].

## Materials and methods

Study design and period

The data presented is part of a two-year, longitudinal, community-based, effectiveness study which was aimed at assessing if consumption of vitamin D-fortified foods (milk and oil) was effective in improving serum 25OHD concentrations and vitamin D status of families. This study was carried out in Pune which is located in Western India (18°N 73°E). In the current study, we present cross-sectional data collected at the baseline of the longitudinal study from October 2020 to November 2020.

Sampling technique

Sample size calculation was carried out for the longitudinal study based on the report by Khadgawat et al. to assess the effect of consumption of vitamin D-fortified milk in Indian adolescents in 2013 [8]. We computed a sample size of 157 subjects per study arm (two arms) with a power of 0.8 at a 0.05 level of significance. We factored in a sample attrition rate 20% per year to obtain a final sample size of 440 participants (220 participants/arm). We enrolled 231 participants (57 families) and 217 participants (55 families) in the fortified and the unfortified arm, respectively. Families were selected by simple random sampling technique. Research staff comprising community health workers and social workers conducted door-to-door screening to identify families eligible for enrolment.

Inclusion and exclusion criteria

Families were considered eligible for enrolment if they comprised two to six members, the entire family consented to being enrolled in the study, and the family was willing to consume the same brand of milk and oil and intended to stay in the same area for the entire study duration. Families were considered to be ineligible to participate in the study if any family member suffered from a chronic condition which affected vitamin D metabolism or if anyone was consuming vitamin D supplements or medications which could interfere with vitamin D metabolism.

Ethics approval

Ethics approval for the study was obtained from the institutional ethics committee (Biomedical and Health Research Ethics Committee, Jehangir Clinical Development Centre Pvt. Ltd.) on July 7, 2020. Prior to enrolment, written informed consent was obtained from all adults and written informed assent was obtained from children seven years and older. This study has been registered with ClinicalTrials.gov (Identifier: NCT05541094).

Data collection techniques

Details on data collection methods have been described previously [9]. We measured the height and weight of all participants using standard techniques. Body fat percent was assessed using the Tanita Body Composition Analyzer (Model MC-780) which works on the principle of bioelectrical impedance analysis. Information on socio-demographic characteristics and sunlight exposure were collected using validated questionnaires. Trained phlebotomists collected venous blood samples which were used for estimation of serum 25OHD_2_ and 25OHD_3_ concentrations by liquid chromatography-tandem mass spectrometry (LC-MS/MS) method on the Waters® ACQUITY™ TQ Detector. Total serum 25OHD was computed as the sum of the serum 25OHD_2_ and 25OHD_3_ concentrations. Vitamin D status was categorized as deficient (< 30 nmol/L), insufficient (30-50 nmol/L), and sufficient (> 50 nmol/L) based on the total serum 25OHD concentrations using the 2011 Institute of Medicine (IOM) guidelines [10].

Statistical methods

Statistical analyses were performed in R (version 4.3.2). Continuous variables have been reported as mean ± SD or median (Q1, Q3), while categorical variables as number (%). The variables were evaluated for normality, and those not meeting normality assumptions were log-transformed. Linear mixed models with family as a random variable were used in the analyses to account for the dependency between subjects in a family. Two separate analyses were performed for modeling: 1) serum 25OHD_2_ and 2) total 25OHD concentrations using family, age, body fat, and sunlight exposure. The strength of the family level component was assessed by calculating the intra-family correlation for each model separately for children and adults using the following formula:

between-family standard deviation^2^ / (between-family standard deviation^2^ + within-family standard deviation^2^)

Higher intra-family correlation (closer the value of correlation to 1) indicated stronger correlations implying that participants were more alike within a family with respect to their serum 25OHD_2_ or total 25OHD concentration. Since serum 25OHD_3_ concentrations contribute significantly to the total serum 25OHD concentrations [9], separate intra-family correlation for these have not been calculated. Instead, they are considered to be accounted for in the intra-family correlation for total 25OHD concentrations.

## Results

Results have been presented for 104 families which included 235 adults and 118 children. Each family had 4 (3, 5) members. Majority of the families (77%) belonged to the middle socio-economic class. The characteristics of the participants have been presented in Table 1. The mean age of the adults was 37.6 ± 2.9 years and that of the children was 11.7 ± 3.7 years. Prevalence of obesity (≥ 25.0 kg/m^2^) was much higher among adults than among children (41% vs 5%, p < 0.05). More than half (63%) of the participants had poor daily sunlight exposure (< 30 minutes).

**Table 1:** Characteristics of the Participants Results have been presented as n (%) for categorical variables and as mean ± SD for continuous variables. BMI: body mass  index. * indicates significantly different from adults (p < 0.05).

Characteristics	Adult (age ≥ 18.0 years)	Children (age < 18.0 years)
n	235	118
Males	96 (41%)	51 (43%)
Females	139 (59%)	67 (57%)
Age (years)	37.6 ± 12.9	11.7 ± 3.7
Height (cm)	157.5 ± 9.1	139.8 ± 18.6
Height for age Z-score	-	-0.5 ± 0.9
Weight (kg)	60.1 ± 14.1	34.9 ± 14.4
Weight for age Z-score	-	-0.6 ± 1.1
BMI (kg/m^2^)	24.2 ± 5.1	17 ± 3.8
Body fat percent	30.8 ± 9.9	17.8 ± 11.2
Nutritional Status		
Normal BMI (18.5 to 22.9 kg/m^2^)	63 (27%)	85 (72%)^*^
Underweight (< 18.5 kg/m^2^)	32 (14%)	12 (10%)
Overweight (23.0 to 24.9 kg/m^2^)	43 (18%)	15 (13%)
Obese (≥ 25.0 kg/m^2^)	97 (41%)	6 (5%)^*^
Sunlight exposure		
Less than 30 mins	152 (65%)	72 (61%)
30-60 mins	38 (16%)	31 (26%)^*^
Greater than 60 mins	45 (19%)	15 (13%)

The serum 25OHD concentrations of the participants have been presented in Table 2. The serum 25OHD_3_ and total serum 25OHD concentrations were significantly higher in adults than in children.

**Table 2:** Serum 25OHD Concentrations of the Participants Results have been presented as median (Q1, Q3). * indicates significantly lower than adults (p < 0.05). 25OHD: 25-hydroxyvitamin D.

Serum 25OHD Concentrations (nmol/L)	Adults (age ≥ 18.0 years)	Children (age < 18.0 years)
25OHD_2_	5.3 (3.7, 7.1)	4.6 (3.3, 7.1)
25OHD_3_	34.8 (20.3, 51.0)	27.7 (15.6, 43.3)^*^
Total 25OHD	40.1 (24.8, 56.7)	33.0 (21.7, 48.4)^ *^

**Figure 1:**
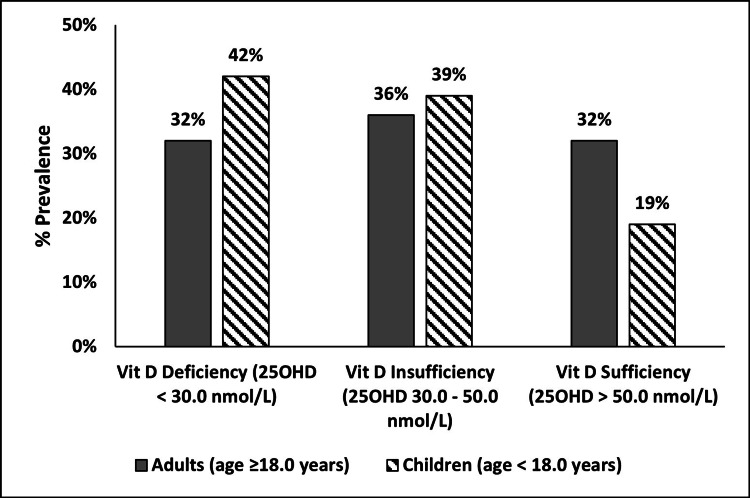
Vitamin D Status of the Participants 25OHD: 25-hydroxyvitamin D.

The vitamin D status of the participants has been illustrated in Figure 1. Around one-third of the participants, both adults and children, were found to be vitamin D deficient. Prevalence of vitamin D sufficiency was significantly lesser in children as compared to adults (19% vs 32%, p < 0.05).

**Figure 2:**
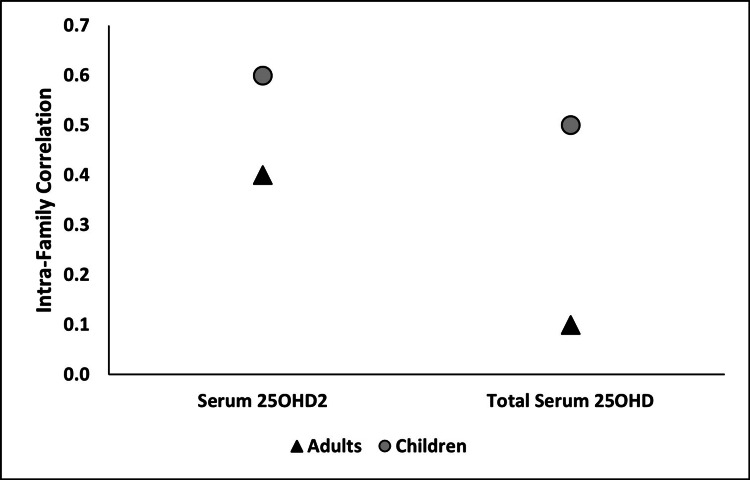
Intra-familial Correlation for Serum 25OHD2 and Total 25OHD Concentrations 25OHD: 25-hydroxyvitamin D.

The intra-family correlation among adults and children for serum 25OHD_2_ and total 25OHD concentrations has been depicted in Figure 2. Intra-family correlation was found to be much higher in children than in adults for both serum 25OHD_2_ (0.6 vs 0.4) and total serum 25OHD (0.5 vs 0.1) concentrations. Also, the correlations for serum 25OHD_2_ and total 25OHD concentrations were similar for children (0.6 vs 0.5) but considerably different for adults (0.4 vs 0.1).

## Discussion

In our study among families residing in Pune, India, we found around a third of the participants to be vitamin D deficient, with the prevalence of deficiency being higher among children than among adults. Intra-family correlations for serum 25OHD_2_ and total serum 25OHD concentrations were found to be stronger among children than among adults. This implies that there is a greater likelihood that within a family, children will have similar serum 25OHD concentrations as compared to the adults. These results suggest a role played by either genetic factors or possibly shared dietary and lifestyle habits among children in a family, thus resulting in a stronger correlation.

In our study, we found vitamin D concentrations to be higher in adults than in children. Consequently, the prevalence of vitamin D sufficiency was significantly greater, and the prevalence of vitamin D deficiency was lower in adults than in children. This finding is similar to results reported by Selvarajan et al. in their systematic review published in 2017, specifically that middle-aged Indians were found to have higher vitamin D concentrations than Indian adolescents [11]. However, our findings are contradictory to those reported by Sreenivasulu et al. among residents of the city of Jodhpur, Rajasthan, who found greater vitamin D insufficiency among the adults than among the children [12]. The reasons for these contradictory findings could be the seasonal variations between Pune and Jodhpur with the latter having arid, hot desert-type climate, while the former has tropical wet and dry climate [13,14]. Also, cultural practices like the *Ghoonghat* (form of veiling) common in Jodhpur may be responsible for greater vitamin D deficiency (VDD) among adult female residents of Jodhpur. VDD was noted in 36% of the participants in our study which is considerably lower than that reported in other studies from India [1,12,15,16]. One explanation for this is the difference in guidelines used for vitamin D status categorization. In the present study, we have defined VDD as serum 25OHD concentrations < 30 nmol/L based on the IOM guidelines [10], while studies have defined a 25OHD concentration of < 50 nmol/L as VDD based on the Endocrine Society guidelines [17].

As evidenced by the literature, this is the first study to report intra-class correlations for serum 25OHD_2_ and total serum 25OHD concentrations by considering the family membership among Indians. The intra-family correlation for serum 25OHD_2_ concentration in children was one and a half times that of adults (0.6 vs 0.4). For total serum 25OHD concentrations, the intra-family correlation was five times that of adults (0.5 vs 0.1). This implies that children within a family were more alike with respect to their serum 25OHD_2_ and total 25OHD concentrations as compared to adults. These findings indicate the role of shared genetics and habits among siblings in a family leading to a stronger correlation. Genetic contribution to vitamin D concentrations has been demonstrated in several studies [18,19]. Also, genetics influence skin pigmentation which is reported to be negatively associated with vitamin D production [20]. While parents are genetically distinct individuals, siblings share genetic traits inherited from both parents. This shared genetic makeup may explain the similarities in vitamin D concentrations observed among children within the same family. Environmental influences, predominantly sunlight exposure, also play an important role in determining vitamin D concentrations and can outweigh genetic influences [21]. Thus, similar daily routines among siblings, such as time spent outdoors playing or in school may also have contributed to the similar vitamin D concentrations noted among siblings in a family.

Furthermore, the difference between children and adults in the intra-family correlation for total 25OHD (0.5 vs 0.1) was much greater than that for serum 25OHD_2_ (0.6 vs 0.4). Given that serum 25OHD_2_, i.e., ergocalciferol, is obtained solely from dietary sources, while serum 25OHD_3_, i.e., cholecalciferol (which is a major contributor to total 25OHD concentrations [9]), is obtained via cutaneous photoproduction as well as dietary intake, the findings seem to suggest that children and adults in a family have similar dietary habits but different lifestyle habits (such as sunlight exposure and time spent outdoors). Finally, among adults, the correlation for serum 25OHD_2_ was four times that of total 25OHD (0.4 vs 0.1) which seems to suggest that chances of shared dietary habits among adults in a family is much greater than that of shared lifestyle habits, thus resulting in poorer correlation for total serum 25OHD concentrations.

There are limited reports available which discuss family correlation of 25OHD [5,6] and no reports on the family correlation of serum 25OHD_2_. Compared to the Danish families who participated in a study by Madsen et al. in 2014, the children in our study had comparable intra-family correlation (0.42 vs 0.5) for total serum 25OHD, while the adults in our study had lower correlations (0.24 vs 0.1) [6]. We have not presented total correlations for all participants taken together because for adults, we used body fat percent values as one of the dependent variables in the linear mixed model used to derive intra-family correlation, while for children, we used body fat percent z-scores. In the study by Robinson et al., we are unable to compare the intra-family correlation for children because it has not been presented, but the correlation for adults was higher than what we observe in our study (0.23 vs 0.1) [5]. Even though the method of 25OHD assessment used in our study (LC-MS/MS) varies from that used by Robinson et al. (chemiluminescence immunoassay) [5], the result is similar in that Indian adults had a lower intra-family correlation as compared to adults in families in Denmark [6] and Mesoamerican countries [5]. Farzin and Dastgiri in their study among Iranian families residing in north-west Iran examined familial aggregation of vitamin D deficiency and not intra-family correlation for 25OHD concentrations [22]. They report aggregation of vitamin D deficiency among brothers, sisters, and spouses [22].

## Conclusions

We conclude that family relationship has an impact on total serum 25OHD concentrations of individuals residing in Pune, India. Our findings indicate that children in a family have stronger intra-class correlation for total 25OHD concentrations than adults. This implies that the likelihood of having similar vitamin D concentrations is higher among children as compared to adults in a family. Thus, if one child in a family is found to have inadequate vitamin D concentrations, measures can be taken to treat or prevent vitamin D deficiency in the other siblings.
